# Using Uncertainty and Sensitivity Analyses in Socioecological Agent-Based Models to Improve Their Analytical Performance and Policy Relevance

**DOI:** 10.1371/journal.pone.0109779

**Published:** 2014-10-23

**Authors:** Arika Ligmann-Zielinska, Daniel B. Kramer, Kendra Spence Cheruvelil, Patricia A. Soranno

**Affiliations:** 1 Department of Geography, Michigan State University, East Lansing, Michigan, United States of America; 2 James Madison College, Michigan State University, East Lansing, Michigan, United States of America; 3 Department of Fisheries and Wildlife, Michigan State University, East Lansing, Michigan, United States of America; 4 Lyman Briggs College, Michigan State University, East Lansing, Michigan, United States of America; University Toulouse 1 Capitole, France

## Abstract

Agent-based models (ABMs) have been widely used to study socioecological systems. They are useful for studying such systems because of their ability to incorporate micro-level behaviors among interacting agents, and to understand emergent phenomena due to these interactions. However, ABMs are inherently stochastic and require proper handling of uncertainty. We propose a simulation framework based on quantitative uncertainty and sensitivity analyses to build parsimonious ABMs that serve two purposes: exploration of the outcome space to simulate low-probability but high-consequence events that may have significant policy implications, and explanation of model behavior to describe the system with higher accuracy. The proposed framework is applied to the problem of modeling farmland conservation resulting in land use change. We employ output variance decomposition based on quasi-random sampling of the input space and perform three computational experiments. First, we perform uncertainty analysis to improve model legitimacy, where the distribution of results informs us about the expected value that can be validated against independent data, and provides information on the variance around this mean as well as the extreme results. In our last two computational experiments, we employ sensitivity analysis to produce two simpler versions of the ABM. First, input space is reduced only to inputs that produced the variance of the initial ABM, resulting in a model with output distribution similar to the initial model. Second, we refine the value of the most influential input, producing a model that maintains the mean of the output of initial ABM but with less spread. These simplifications can be used to 1) efficiently explore model outcomes, including outliers that may be important considerations in the design of robust policies, and 2) conduct explanatory analysis that exposes the smallest number of inputs influencing the steady state of the modeled system.

## Introduction

Socioecological systems are perpetually dynamic and nonlinear [1], [2], [3], [4], [5], [6], [7]. To account for this complexity, researchers often employ agent-based models (ABMs). Socioecological ABMs are computational models composed of heterogeneous entities (called agents) that shape a common environment representing an integrated human and natural system [2],[8],[9]. ABM offers a robust vehicle for simulating socioecological systems, for example landscape dynamics, by providing means of representing autonomous and decentralized decision-making that results in emergent landscape-scale characteristics (e.g., land value, land use patterns) and phenomena (e.g., biodegradation, land conservation). For example, ABMs are often used to model agricultural land systems, in which the environment is operationalized by spatial layers (maps) including land use, soil productivity, vegetative cover, and precipitation. The agents, or actors, in the system may include farmers who cultivate their land, developers who buy and sell land parcels, residents who inhabit select locations, and authorities that adopt and enforce land-related policies [10],[11],[12],[13],[14],[15],[16]. As with all modeling of such complex systems, ABMs are inevitably prone to uncertainty reflecting insufficient knowledge of the processes driving these coupled human-natural systems. For example, we have incomplete knowledge on the relationships and feedbacks between crop market fluctuations, farmland management, and temporal dynamics in nutrient cycling affecting such systems, nor do we fully understand the inherent randomness of environmental and social events like the popularity of agritourism during a heat wave. Not surprisingly, comprehensive evaluation of uncertainty has emerged as an important topic of social-ecological research, including environmental modeling [17],[18],[19],[20], land use and land cover change [5],[21],[22], and geographic information science [23],[24],[25],[26]. The need for proper handling of uncertainty also has been widely recognized in ABMs of socioecological systems [27],[28],[29].

**Figure 1:**
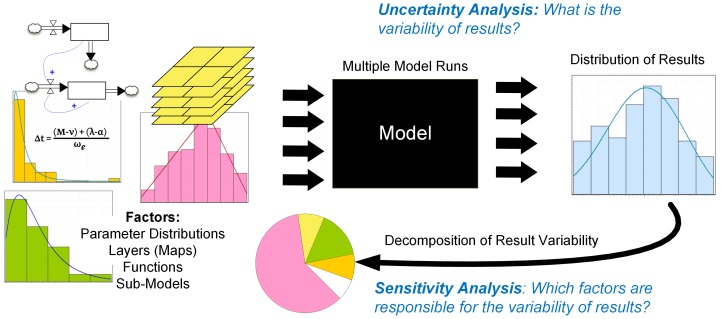
Uncertainty and sensitivity analyses of model output.

We argue that systematic evaluation of ABM uncertainty should comprise a joint quantification of model output variability and its sensitivity to inputs (called factors) - Figure 1 [5],[21],[28],[29],[30],[31]. Consequently, we propose a new framework for evaluating uncertainty of ABMs. We demonstrate how an integrated quantitative uncertainty analysis and sensitivity analysis (UA-SA) can be employed in ABM development to meet three modeling objectives: 1) to evaluate the validity of simulation results (using uncertainty analysis - UA); 2) to generate a more parsimonious model (using sensitivity analysis - SA), and 3) to prioritize input data refinement by identifying the ABM factors that are mostly responsible for model output variability (using both UA and SA). Factors comprise various uncertain model components including variables, parameters, spatial data (maps), functions, and sub-models that jointly influence ABM results - Figure 1 [32]. Uncertainty analysis (UA) evaluates how the variability of factors propagates through the model and affects the variability of output values. The objective of UA is to quantify the distribution of results given uncertain factors. Conversely, sensitivity analysis (SA) evaluates how factor variability contributes to model output. Although ABMs are relatively common in socioecological research, studies rarely include the joint use of quantitative UA-SA, suggesting that these stages of ABM evaluation are perfunctorily, if at all, undertaken [33],[34],[35].

Recognized as important for scientific understanding, quantitative UA-SA has been employed in a number of non-ABM studies on socioecological systems. Examples include ecological modeling of ecosystem vulnerability to climate change [36], hydrology and water use [37],[38],[39],[40], species interactions and community stability [41], changing human environmental attitudes [42], water eutrophication [43], and coral reef degradation [44]. In ABMs studies, the most common approach involves an UA that summarizes the results of Monte Carlo simulation based on simple random sampling or, in the case of SA, running the model with extreme values of its factors or using a limited number of values, with little or no quantification of the influence of these factors on the variability of results [1],[45],[46],[47],[48]. One possible explanation for the lack of quantitative UA-SA in socioecological ABM is the relative infancy of the AB methodology coupled with the flexible protocol for executing AB simulations. There is a need for a well-defined UA-SA framework tailored to meet the specific needs of ABM, such as handling the very large number of heterogeneous factors (at least one per agent) of a highly nonlinear model.

Quantitative UA-SA in ABMs can serve many purposes [31],[49],[50],[51]. It can be used to strengthen trust in model realism and to eliminate model factors that have negligible influence on the variability of the output, allowing for a simpler, easier to understand model. Therefore, UA-SA together provide information on influential factors that significantly affect the variability of model results. They allow scientists to gain a deeper understanding of the complexity of the model, its uncertainties, interrelationships, and its potential future scenarios. UA-SA provide a means of asking ‘what if?’ questions that help to validate or disqualify the results [52]. UA-SA should be included in all ABM exercises using methods that systematically examine model factors and outputs to build credible models necessary for addressing problems at the science/policy interface [53],[54]. We argue that a reliable simulation-based policy analysis requires simplified yet practical models, and that integrated quantitative UA-SA associated with ABM of socioecological systems is essential for scientific understanding for two reasons. First, uncertainty is a fundamental property of complex systems that cannot be ignored. Moreover, because a portion of this uncertainty is irreducible, a socioecological model that generates results with little or no variability has little practical value. Second, a distribution of UA outputs, including the tails and means, provides an opportunity for *exploration* of extreme system behavior. Although highly unlikely, boundary cases may result in radical changes of significant consequence to society and/or the environment. On the other hand, scientific *explanation* requires considerable accuracy, which can be achieved through reducing output variability in order to improve model performance.

In the next sections, we describe how quantitative UA-SA can be used to build ABMs for policy analysis and exploration. While still nascent, a comprehensive UA-SA have been applied to ABMs in a few previous studies [28],[30],[35],[55],[56],[57]. For example, Fonoberova et al. [35] use UA-SA to identify factors for model reduction in an ABM of criminal activity, while Parry et al. [55] use UA-SA to identify the highly sensitive factors that need further refinement in an ABM of bird population. What sets our manuscript apart is the use of comprehensive UA-SA separately for model explanation and exploration, by focusing on the refinement of the most influential factors (explanation) and the reduction of the least influential factors (exploration) of a socioecological ABM. We employ variance decomposition of the ABM output - a method commonly used in ecological modeling outside of ABMs [58],[59],[60]. UA is applied to build a legitimate model, where the distribution of model results informs us not only about the expected value validated against independent data, but also provides information about the spread around the expected value and the extreme (boundary) results. SA is then employed to produce a parsimonious model. Two cases are examined. First, we build a practical *exploratory model* that allows scientists to simulate low-probability but high-consequence events that may be of high policy relevance. Second, we build a more *explanatory model* that provides the means of describing the system with higher accuracy. Specifically, we postulate that the explanatory power of a model lies in illuminating the core underlying processes [61] and exposing system-wide regularities [62], which manifest themselves through the mean of the output of interest. The proposed framework is applied to the problem of modeling farmland conservation and resulting land use change (from agriculture to fallow), demonstrating the utility of UA-SA for contributing to science and policy.

## Materials and Methods

### Uncertainty analysis

UA produces a distribution of model results (Figure 1). It requires multiple model runs, where factor values are randomly chosen from their respective distributions. Because the results of quantitative UA-SA are computationally expensive, the selection of the sampling method used to perform UA is essential. Following Saltelli et al. [63], we use quasi-random sampling that generates samples more uniformly over the entire factor space than simple random sampling. A sample is then used to execute the model, which produces an individual output value. In our case study, for example, the output is a measure of total area of land converted from agriculture to fallow. These results build a distribution of outputs that can be further analyzed using descriptive statistics. Two statistics are particularly useful: the mean that represents the central tendency of the stochastic process, and the variance that summarizes the variability of the results. Variance is then used as input to SA (UA is, therefore, a prerequisite to SA).

### Sensitivity analysis using output variance decomposition

Commonly, SA involves modifying the value of one factor (while keeping the other factors constant) and observing the effects of this change on model results. This method, referred to as one-parameter-at-a-time (OAT) [33],[64],[65], is most often used by socioecological modelers. The prominent examples, closely related to socioecological ABMs, include the use of OAT in land use change cellular automata models to evaluate their sensitivity to map resolution and the size and configuration of neighborhoods [66],[67] and the use of OAT to identify the most sensitive factors in an epidemiological ABM of the spread of measles among humans [68]. OAT popularity may be attributed to its simplicity, low computational cost, clear starting point in the form of a baseline parameter set, and the fact that the observed changes in outputs can be easily traced back to changes in specific factors [33]. Unfortunately, the utility of OAT for complex socioecological ABM is limited. The arbitrary choice of which factor to modify and by what amount is problematic when the magnitude of key system drivers is hard to determine [28]. Also, OAT does not explore the variability of factors in combinations and, consequently, assumes a linear relationship between inputs and outputs. Finally, OAT is of limited use in exploratory modeling, because it does not test the full range of factor variability and therefore minimizes our ability to simulate extreme, but catastrophic, events. As an alternative to OAT, we utilize a global SA approach, which is based upon simultaneous perturbations of the entire model factor space, examining the factors both individually and in combinations [69],[70].

Our global SA uses model output variance decomposition in which the variability of the area of fallow land (resulting from farmer agent decision making) is decomposed (partitioned) and distributed among model factors evaluated in various combinations [69],[71]. Factor sensitivity is quantified using two measures referred to as a first order sensitivity index, S, and a total effects sensitivity index, ST [69],[70],[72]. Index S measures the independent, fractional contribution of each individual model factor to output variance. The ST index estimates the overall contribution of a given factor to output variance including its interactions with other factors. Assuming that model output Y has unconditional variance V, the indices of a given factor (i) are formalized as follows:
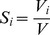
(Eq. 1)

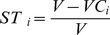
(Eq. 2)Where V_i_ is the variance of Y due to the variability of factor *i* alone, and VC_i_ is the conditional variance due to all model factors except *i*. The sum of all S indices (ΣS) is the fraction of output variance that can be explained by the individual factors alone. Therefore, the formula:
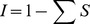
(Eq. 3)gives the fraction of output variance due to the interactions (*I*) between the factors. This succinct measure of interactions can be further analyzed using the ST indices, which provide information about the total (first and higher order) influence of each factor on output variance. For more details on variance decomposition, the reader is referred to Saltelli et al. [70], Lilburne and Tarantola [69], and Homma and Saltelli [72], among others.

**Figure 2:**
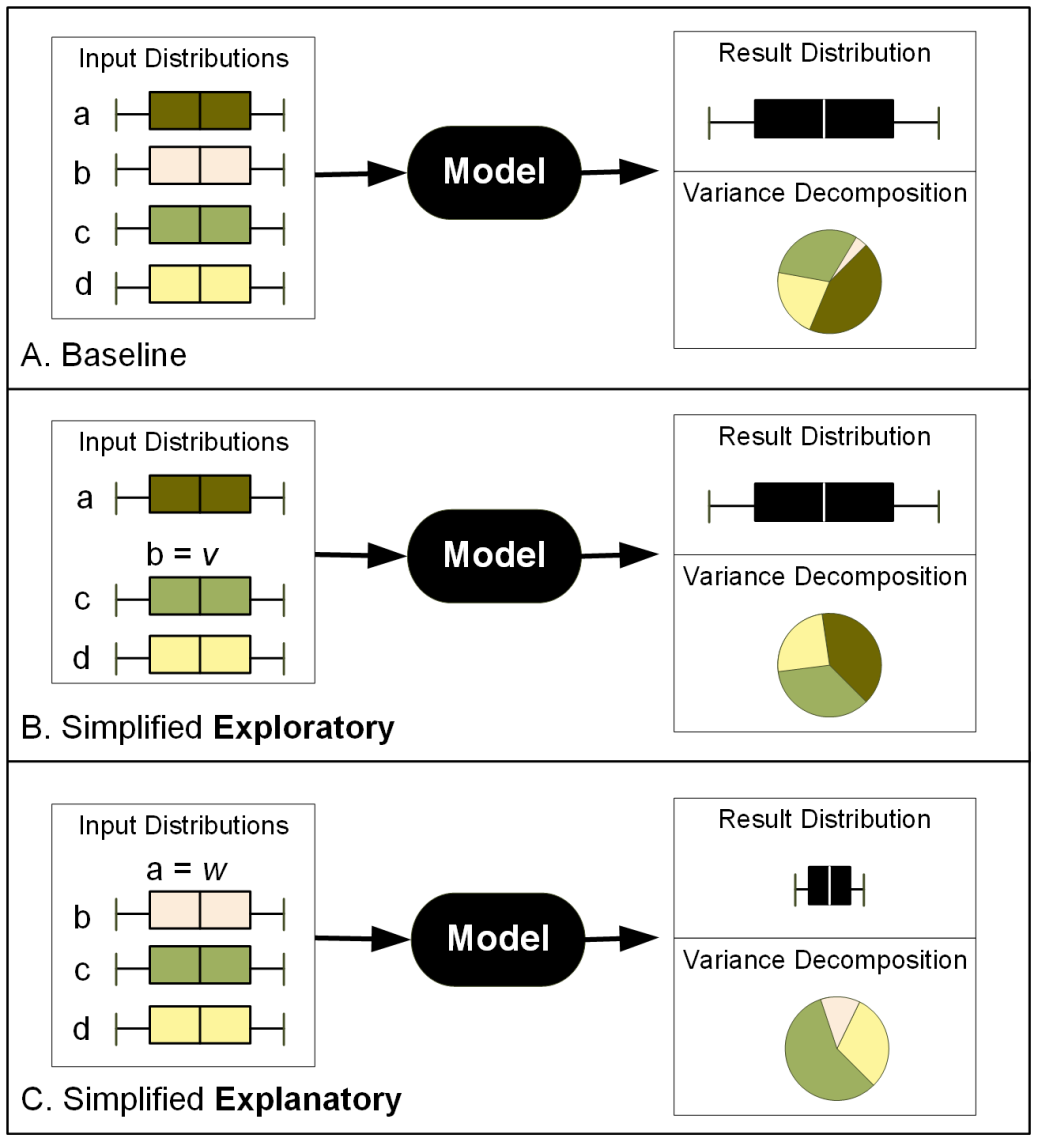
A framework for uncertainty and sensitivity analysis of ABMs of socioecological systems. Applying variance decomposition to simplify a stochastic model (A), and maintain its exploratory power embodied in outcome variability (B) or improving its explanatory power by reducing its outcome variability (C).

The (S,ST) pairs are quantified as ratios of the conditional output variances to the total variance and, thus, measure the relative contribution of each factor to output variance (Figure 2A). Factors with relatively high S (ST) values will have the greatest impact on the variability of model results. When these factors are refined or fixed to constant values, the result is a reduction in output variance. We use this property of the (S,ST) pairs to operationalize the *explanatory power* conception of modeling (Figure 2B). The major premise of model explanatory power is that additional observations used for estimating the most influential factors get us closer to an accurate representation of the underlying system. By better approximating values of the most influential factors, especially in cases where these factors dominate the output, we can unravel the interrelationships among other factors and expose model nonlinearities. Conversely, if we fix factors that have S (ST) values close to zero (i.e. the non-influential factors), we do not significantly change the variance of the results. Instead, we derive a simplified model with quantitative *exploratory power* (embodied in variance of a given output) equal to this model's baseline implementation (Figure 2C).

### Case study: ABM of Michigan farmer enrollment in the Conservation Reserve Program

We use quantitative UA-SA for land use model simplification and factor prioritization. The goal is to build a simpler representation of an ABM with two distinct objectives: policy analysis that would benefit from exploratory modeling [73], and advancing science through explanatory modeling [74]. Our case study considers the participation of farmers in a land conservation program aimed at protecting ecologically valuable areas.

**Figure 3:**
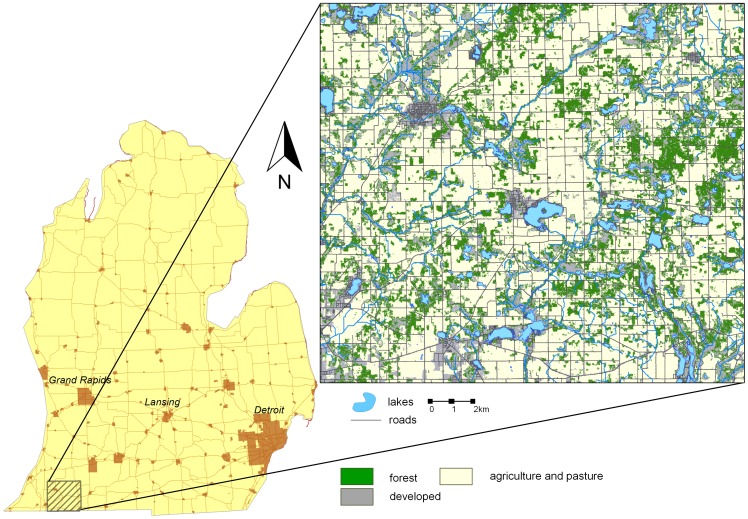
Study area in Michigan, U.S.

Published research suggests that farmers' decision to participate in a land conservation program is driven by both financial and nonmonetary drivers [75],[76],[77],[78]. These findings are based on conventional statistical analyses of survey data. Few studies have explicitly modeled the decision processes and analyzed the resulting spatial configurations of conserved land [16]. Following this observation, we develop an ABM of agricultural land conservation decision making. The model simulates voluntary participation in the U.S.'s largest land protection program, the Conservation Reserve Program (CRP) [79]. We examine CRP enrollment in southwest Michigan, U.S. (Figure 3). The area covers 985 square km, with 2687 farmland parcels. This area is characterized by large proportions of agricultural land with about 3% of farmland enrolled in CRP according to the U.S. 2007 agricultural census [80].

### Model description

**Figure 4:**
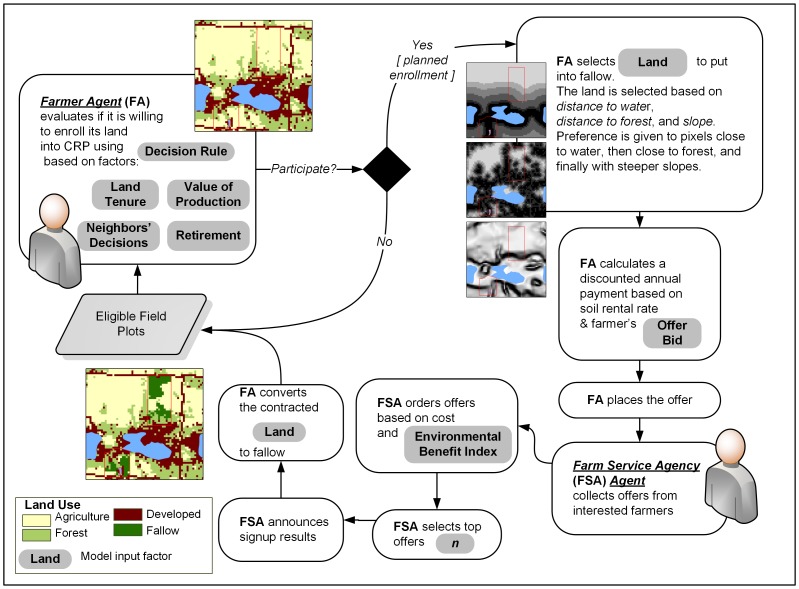
Agent-based model of enrollment in Conservation Reserve Program.

In the ABM reported herein, CRP enrollment is simulated based on well-defined federal regulations [79]. Two types of decision makers are involved in this process (Figure 4): [1] farmers who decide whether or not to participate in CRP and [2] the Farm Service Agency (FSA), which evaluates, selects, and accepts farmer enrollment offers. The basic spatial unit of CRP decision-making is a farmland parcel. During the model setup, a farmer agent (FA) is associated with various socio-demographic and economic factors (*land tenure*, operator's *retirement*, and the *value of production* on a farm; described in later sections). The FA is then assigned to a parcel.

As a first step, the FA calculates their willingness to enroll in the CRP based on decision criteria (factor values) including financial motives and nonmonetary drivers. To calculate the willingness to enroll, we apply a group of aggregation operations (aka *decision rules*) called Ordered Weighted Averaging - OWA [81],[82]. OWA allows for simple representations of different conceptions of risk related to CRP enrollment, which, after acceptance, is mandatory for at least ten years. OWA decision rules range from the most risk-averse, where values of all decision criteria must be positive, to the most risk-taking where only one decision criterion needs to be nonzero. For example, if an agent makes a decision to participate in the program based on low value of production AND retirement, that decision is risk-averse, whereas if the decision is made based on low value of production OR retirement, the agent is risk-taking. Agent's willingness to enroll is extended with a simple group interaction mechanism, in which farmers adopt imitative behavior [83] based on the decisions made by other proximal farmers. An FA incorporates into its decision mechanism the *density of enrollment* in its neighborhood, measured as the ratio of CRP-enrolled neighbors to the total number of neighbors within 0.5 km from the parcel.

If an FA's willingness to enroll exceeds an empirically derived threshold, the agent selects a *fraction* of its parcel for potential enrollment [77]. Eligible sites in the parcel (pasture and cropland) are rank ordered based on distance to water, distance to forest, and land slope, and the first *fraction* of sites is selected. Next, the FA builds an offer by calculating an expected annual payment based on soil rental rates [84]. To increase its offer competitiveness, the FA reduces the payment using a *bid* rate established by USDA [85] and estimates a discounted annual payment (DAP). The FSA agent collects offers from the FAs and selects a subset (*n*) of them based on the *environmental benefit index* (described in the following section), the signup budget, and the DAP. Next, FSA announces the signup results leading to land use change from agriculture to fallow. In sum, the location and area of fallow land results both from the FAs' decisions to participate and the FSA's decision to accept their offers. The process of CRP signup is repeated annually for ten years (minimum CRP contract length). Land use change maps constitute the output of the model. They are summarized into the *total area of fallow land*. This scalar is used in the UA and SA.

### Model input data

**Figure 5:**
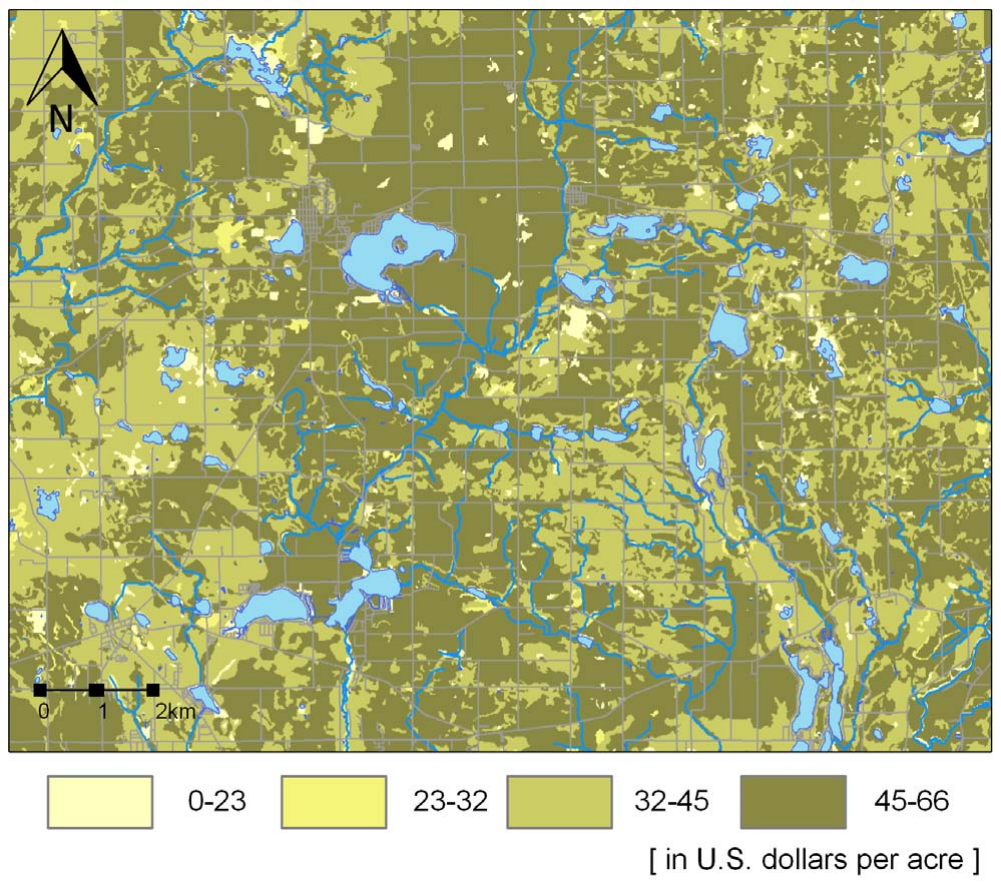
Soil rental rates (the southeast fragment of the study area).

The ABM uses a number of factors, some that are readily available and some that we derived from auxiliary resources, including land uses obtained from 2010 cropland raster layers [86], freshwater ecosystems from a lakes and rivers geodatabase [87], soil data [88], and slope [89]. The major geoprocessing operations were mapping land eligible for CRP [79], deriving spatial layers that influence FA's choice of area to enroll (Figure 4 - distance to water, distance to forest, and land slope), and generating the soil rental rate (SRR) and the environmental benefits layers. The SRR layer (Figure 5) was derived from a soil productivity index map for the State of Michigan [90] and county cash rental rates [84].

### Deriving environmental benefits

Ranking of CRP offers is based mainly on their Environmental Benefits Index (EBI) values. EBI is a composite index based on multiple rated criteria describing benefits for wildlife, water quality, soil erosion, long term maintenance of installed vegetation, and air quality [85]. To optimize environmental benefits per dollar expended for rental payments, the EBI is adjusted by a cost and bid rating scale. Offers with lower total annual payments and higher bids (voluntary reduction by the farmer of the offer value below the maximum payment) receive highest priority.

**Figure 6:**
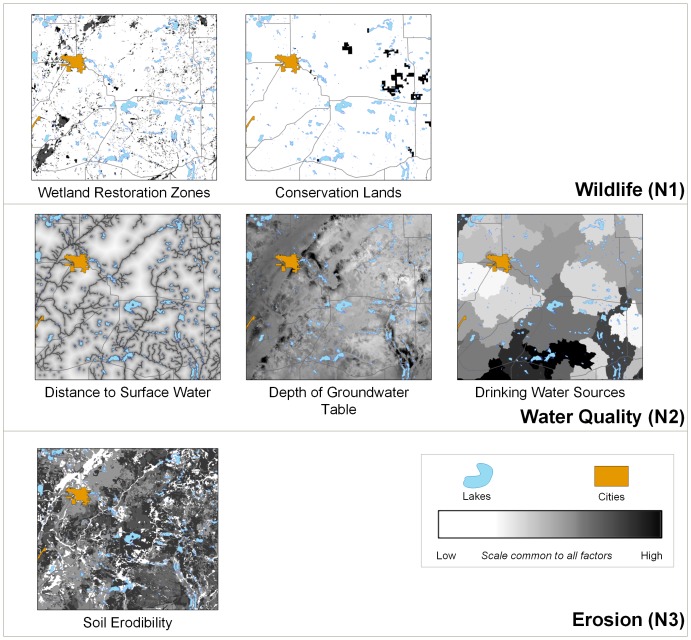
Benefit layers used to calculate six composite EBI surfaces. Each EBI surface is a sum of one of the N1 layers, one of the N2 layers, and the N3 layer. All N1, N2, and N3 layers are standardized based on their respective point scales [78]. The remaining benefit criteria used in EBI calculation (vegetation and air quality) were not used due to their negligible role in the area of study.

EBI can be quantified in many different ways, resulting in substantial uncertainty. Consequently, we used alternative conceptions of the benefits (Figure 6), weighed by their respective point scores [85] in various combinations to generate six different benefit layers used interchangeably in the ABM. The values of EBI range from 50 to 350 points and the six alternative EBI surfaces have moderate to high positive correlation (min Pearson's r = 0.35, max Pearson's r = 0.89).

### Farmer's decision to participate

Statistical and econometric studies of CRP enrollment point to five major categories of independent variables used to predict participation in land conservation programs: farm, household, and environmental characteristics; government assistance; and farmers' attitude and perception [77],[78],[91],[92],[93],[94]. We used USDA's Agricultural Resource Management Survey (ARMS) [95], a semi-annual survey of American farming businesses and households sponsored by USDA's Economic Research Service and the National Agricultural Statistical Service, as our data source for farm finances, production practices, and household characteristics.

We developed an *a priori* set of candidate multiple regression models to understand farmer participation in the CRP based on results of prior studies about farmer participation in land conservation programs. Using a multimodel inference analytical approach based on the Akaike information criterion (AIC), models with relatively low AIC values were considered the most parsimonious, balancing bias and variance of model predictions [96]. We assigned relative strengths of evidence to each candidate model according to AIC weights and evaluated explanatory variables in terms of deviance explained. From this process, farmers' retirement status (RETIREMENT), the total value of farm production (PRODUCTION), and the ratio of farmed to owned acres (TENURE) received the most support in terms of deviance explained (Table 1). Consequently, these three attributes became FA's decision criteria (Figure 4). Finally, the dependent variable used in the regression models (yes/no CRP participation decision) was used to estimate the threshold for FA's willingness to enroll equal to 0.87, which reflects the ratio of farmers enrolled in CRP to total farmers in 2010 in Michigan based on the ARMS data.

**Table 1:** Probability distributions for factors used in ABM simulations.

Factor Name	Factor Description	Probability Density Function
RETIREMENT	Primary operator retired from farming (0 -retired, 1- working).	D = {(0,.06), (1,.94)}
PRODUCTION	Total value of production on a farm (normalized).	D = {(0,0), (.2,.06), (.4,.06), (.6,.11), (.8,.15),(1,.62)}
TENURE	Ratio of owned to operated acres.	D = {(0,.04), (.2,.14), (.4,.18), (.6,.14), (.8,.15),(1,.35)}
DE	Extent of FA's neighborhood used to calculate the density of enrollment in FA's geographic vicinity.	U = {.5 km to 1.5 km with increments of 100 m, with equal probability of selection}
OWA	FA decision rule based on ordered weighted averaging, with varying attitudinal character i.e. the level of “orness” [81].	D = {17 combinations with equal probability}
LAND	Fraction of parcel to set aside for conservation.	U = (0, 1]
BID	Voluntary reduction by the farmer of the offer value below the maximum payment rate.	D = {0% to 16% of offer reduction with increments of 1, with equal probability of selection}
EBI	Environmental benefits index dataset.	D = {6 layers with equal probability}
n	Number of offers (contracts) accepted annually by FSA.	D = {18 to 28 with increments of 1, with equal probability of selection}

U - uniform distribution, D - discrete distribution (value, probability). All factors were normalized to [0.0, 1.0]. All data are for CRP sign-up 41 in 2010 [108].

### Factor distributions

Given the CRP enrollment procedure and the available data, we identified nine factors for the ABM. Seven factors are attributed to the FA and the remaining two to FSA (Figure 4). The three independent explanatory variables used by the FA in the enrollment decision (land tenure - TENURE, value of production - PRODUCTION, and operator's retirement status - RETIREMENT) were included as individual, farmer-level factors in the form of empirically-derived probability density functions - PDFs (Table 1). These functions epitomize the financial and nonmonetary drivers affecting the FA's land conservation decision.

Empirical data for other factors were only partially available. Consequently, we used a uniform PDF for the other model factors [97]. The density of enrollment in the FA's neighborhood depends on how the neighborhood is defined. In our model, we delineated neighborhood based on distance from FA's parcel, which varied from 500 to 1500 m. For OWA, we assumed various magnitudes of attitude to risk, where each level had an equal probability of selection. To select the fraction of land for potential enrollment (LAND in Figure 4), we assigned a uniform distribution from 1% to 100% of parcel area (partial to full land enrollment). The number of offers accepted by FSA was based on the budget allocated to CRP per county [98].

### Design of experiments

Our simulations include three computational experiments. In experiment one (EXP1, 2560 model runs), our base scenario (Figure 2), we run Monte Carlo simulations using all nine factors. In experiment 2 (EXP2, 1536 runs), the simplified exploratory scenario, we include only those factors that most influence the variability of the total area of fallow land (AREA), calculated at the end of model execution. The simplified explanatory scenario with variance reduction is implemented in experiment 3 (EXP3, 2304 runs), where we fix the value of the most influential factor from EXP1, leaving the remaining factors unchanged. All simulations were run using high performance computing at Michigan State University. Factor samples were produced using the quasi-random Sobol' experimental design [99], which is the most optimal method to approximate the values of the S and ST indices [63],[69]. The ABM was implemented in the Python programming language (http://www.python.org/) and the (S, ST) indices were computed using the SimLab software package for uncertainty and sensitivity analysis (http://ipsc.jrc.ec.europa.eu/?id=756). Statistical regressions were completed utilizing R, version 3.0.2.

## Results

**Figure 7:**
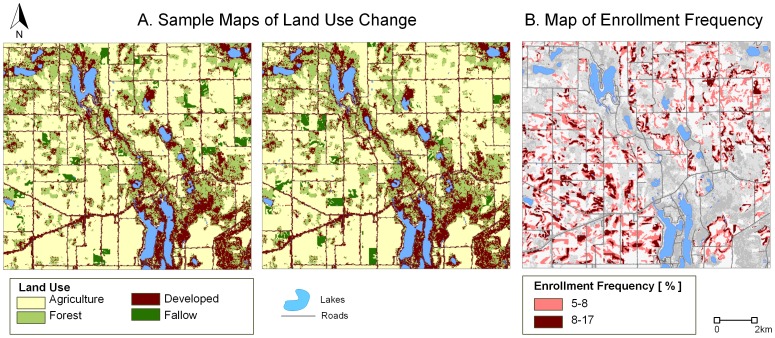
Example output land use maps (A), and the frequency of agriculture-to-fallow conversion (B). For clarity, only the southeast portion of the study area is shown.

The results of our ABM simulations are land use maps with one additional category, *fallow land*, when compared to our input land use maps. Example results from EXP1 are depicted in Figure 7A. Because FAs make decisions on a site-by-site basis, most of the parcels enrolled in CRP at the end of model execution have only a portion of their land enrolled in CRP. Figure 7B illustrates the frequency of site enrollment (number of times a site is enrolled for all ABM executions). Note the considerable spatial variability in site enrollment. Most sites are only selected 5–8% of the time. We hypothesize that this dispersed enrollment is caused by the complex interactions between the nine factors. We utilize UA-SA to illuminate these complexities and focus on the causes of CRP enrollment variability.

### Uncertainty analysis

**Figure 8:**
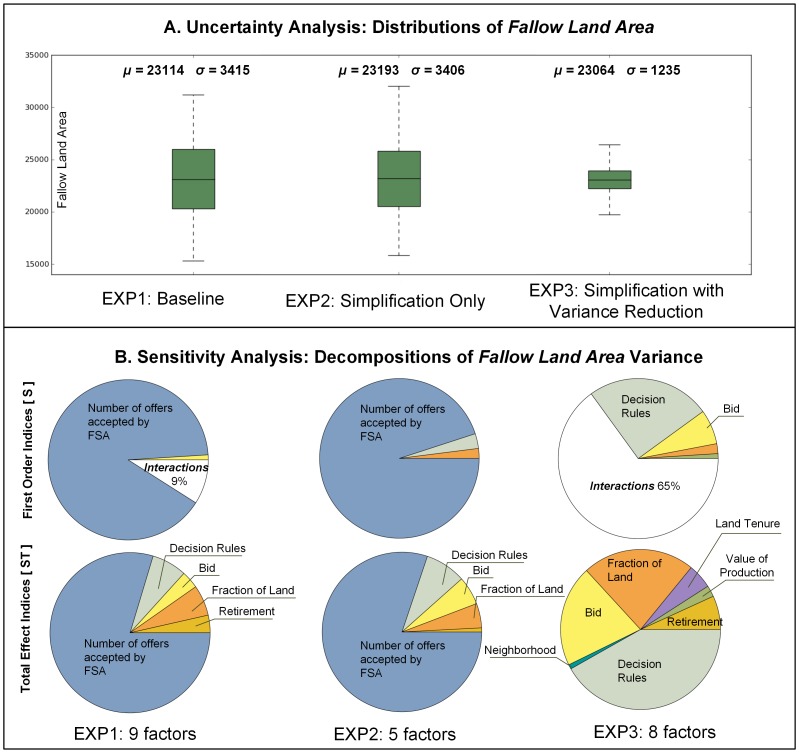
Results of uncertainty (A) and sensitivity (B) analysis for the output variable fallow land area. Fallow land area is reported in map units (equivalent of 30 m). Factor labels used in text: number of offers accepted by the Farm Service Agency - n, payment reduction used by the farmer agent to increase offer competitiveness - BID, FA's decision rule - OWA, fraction of farmland enrolled in CRP - LAND, FA's retirement status - RETIREMENT, FA's value of production - PRODUCTION, land tenure - TENURE, density of enrollment in the neighborhood - DE, measurement of environmental benefits - EBI, factor interactions - I (Equation 3).

To explore the variability of CRP enrollment, we performed UA by examining the distribution of the total area enrolled in CRP (*total fallow land area* - AREA). Figure 8A summarizes the distributions of AREA for each of the three experiments. We also calculated the mean and variance of AREA per experiment. The mean CRP AREA was between 5120 and 5150 acres, with results of no experiment being significantly different from any other (one-way ANOVA (F(2,6397) = 0.961, p = 0.38), confirming that all ABM representations are equivalent. Since our experimental design uses a more uniform (quasi-random) sampling compared to the typical ABM Monte Carlo simulations that are based on simple random sampling, we can infer that the calculated mean is indeed the true (accurate) measure of central tendency in AREA distribution. Consequently, we can use this value to validate the model against an independent dataset. The U.S. Agricultural Census [80] reported 5490 acres (∼24,700 map units) of CRP land in the study area, which is about 7% more than the mean for the baseline EXP1, rendering the results plausible for further evaluation.

We used the variance to evaluate the degree of AREA variability. As expected, the variances of EXP1 and EXP2 are approximately equal. Consequently, the simplified model used in EXP2 can be used in *exploratory* analysis without the loss of variability necessary when evaluating the CRP policies. Conversely, EXP3 (in which data on the most sensitive factor was refined) produces a distribution much more centered around the mean when compared to the baseline. Consequently, the simplified and refined ABM used in EXP3 can be used in *explanatory* analysis of the social, economic, and ecological processes associated with CRP participation. The following section explains the details on how we arrived at these two ABM simplifications.

### Sensitivity analysis and model simplification

UA alone does not provide any information about the influence of individual factors on AREA variability. Without SA it would be impossible to build the simpler yet equivalent versions of our ABM. By performing the decomposition of AREA variance, we can identify factors in the initial version of the ABM that can be either reduced without the loss of ABM exploratory power (EXP2), or refined if our objective is to explain the processes (EXP3).

Figure 8B shows pie charts of the S and ST indices for all three experiments. Because factors with relatively high values of S have the most effect on the of total fallow land area, we look for factors that, if fixed singly, would most reduce the variance of AREA. In the baseline EXP1, the highest S is recorded for the number of offers accepted by the FSA (n). Trivially then, the extent of farmland conservation is first and foremost driven by the FSA signup choices. Given that CRP is competitive among farmers, the ABM confirms the observation that program participation depends on the federal budget allocated to annual payments. Only about 10% of AREA variance can be attributed to factor interactions, which occur between n, OWA, BID, LAND, and RETIREMENT. Due to their influence, these five factors were included in a simplified version of the model in EXP2 (central box plot and pie charts of Figure 8A and 8B). Because we only excluded factors that had negligible influence on the distribution of AREA (which were set to constant values - either their mean or median), the resulting and baseline distributions are nearly identical, including their means and variances. More importantly, variance decomposition generated S and ST indices consistent with the original model formulation. We can therefore conclude that our ABM formulation used in EXP2 meets the criteria of a simplified *exploratory* model (Figure 2). This simplified model is more efficient computationally - an indispensable feature for models used in policy analysis [73]. At the same time it maintains result variability, which can be of use when identifying the less probable but highly consequential policy scenarios.

In EXP3, we set n = 23 (its midpoint number of offers), to demonstrate how the behavior of our ABM changes when, instead of fixing the negligible factors, we do so for the most influential factor. This scenario imitates a situation in which we obtain more accurate data on the most sensitive factor of the model. There was a significant reduction in AREA (Figure 8A), and although the mean is roughly the same as its initial value, the spread around the mean decreased by 64% compared to EXP1. EXP3 is also characterized by a more complex behavior than the first two experiments. Only 35% of this reduced variance can be explained by individual factors (Figure 8B right). The total effect indices suggest that non-monetary motives (perception of risk and FA's retirement) are equally important in FA's decision as the financial drivers (BID, LAND, PRODUCTION, TENURE). We hypothesize that a portion of these interactions can be attributed to the functional relationships between factors. For example, if the fraction of land to convert in a particular parcel has a relatively high value while the OWA rule is conjunctive (AND-only) [100], a large portion of land has the potential to become fallow. However, if the OWA rule is disjunctive (OR-only), an offer can be accepted (and the land can be set to fallow) even when the fraction of land to convert to fallow is relatively low, provided that the other factors (RETIREMENT, PRODUCTION, TENURE) compensate for LAND and encourage land conservation. In summary, while we reduced the range of the distribution for AREA in EXP3, we also exposed more complex dependency among the remaining factors than initially observed. By “improving” the most influential factor, we illuminated the complexity of FA's decision making. We can therefore postulate that this simplified ABM carries more *explanatory* power than the original model.

### Limitations

Our combined quantitative UA-SA framework serves as a tool for better-informed ABM building. It leads to equivalent but simpler representations of a given socioecological system. Output uncertainty can be greatly reduced if more effort is put into improving the quality of data on the most influential factors (factor prioritization) through additional field studies, surveys, or auxiliary databases. However, the UA-SA framework also has limitations due to two design aspects: factor distributions and the type of output variables used (i.e., the way we measure or assess model results). A different output variable (e.g. the patchiness of fallow land, the cost of vegetation installation, or the long term reduction in nutrient loading to lakes) might point to a different set of influential factors. For example, the use of spatial metrics applied to output land use change maps [101] may lead to alternative explanations of model uncertainty [30]. Similarly, the type and characteristics of the probability distributions used for each factor (e.g. uniform versus normal distribution for LAND) could influence both the variability of outputs and the relative contribution of factors to this variability.

The ABM presented here is of limited use for natural resource management practice. Data for most of the factors are either simulated or come from secondary sources and some of the mechanisms are poorly defined. Future model improvements will require surveys of and interviews with farmers and government officials. The recent decline in CRP enrollment suggests that increasing crop prices and government subsidies may play a significant role in the extent of land conservation [102], indicating the importance of such research. Finally, more insight into the spatial configuration of fallow land (connectivity, clustering, or dispersion of fallow land) may be necessary to better evaluate the ecological benefits of land conservation resulting in the prioritization of protected areas.

## Discussion

ABMs have distinct advantages over other modeling approaches due to their abilities to couple human and natural systems, to incorporate micro-level behaviors among interacting agents, and to understand emergent phenomena due to these interactions. Their use thus far has been primarily by researchers for descriptive and predictive purposes [103]. This fact may explain their limited use in policy-making; ABMs' abilities to make accurate predictions have been questioned [62],[104]. We have addressed this perceived limitation using our quantitative UA-SA approach by identifying and fixing the values of the most influential factors, thereby reducing the variance of model results. Doing so allows researchers to gain a greater understanding of the individual and interactive effects of different model factors. Further, by controlling the factors that explain the most variation in the output, researchers can expose the smallest number of factors that influence the steady state of a system. In our CRP example, we fixed the number of offers accepted by the FSA in our exploratory model (EXP 3), thereby reducing the number of factors by one as compared to the baseline model. Although the mean of our output variable, fallow land area, was essentially the same as that of the baseline, the variance decreased dramatically. Thus, this explanatory model revealed complex and important interactions among the remaining factors.

We also used the quantitative UA-SA approach to improve the ABM's policy relevance. Lempert [105] argued that ABM policy relevance might be improved if utilized for exploratory rather than predictive purposes, reflecting the fact that there is often great uncertainty and little agreement among stakeholders regarding complex, dynamic processes and corresponding decisions. Whereas his suggestion was to exercise large numbers of model runs and use various criteria including robustness, resilience, and stability to evaluate different policies, we have offered a more tractable approach. By identifying the most influential factors and ignoring others, we developed an ABM model for exploratory purposes; a simplified model with no loss in output that allows for the exploration of various policy scenarios, including rare but potentially catastrophic events. In our example, our exploratory model (EXP 2) used only five factors as compared to nine in the baseline model. Yet, the mean and variance of our output variable, fallow land in conservation, changed little from the baseline. Thus, by reducing model factors, we are able to efficiently explore different, policy-relevant scenarios.

Interest in the study of complex socioecological systems or coupled human and natural systems has risen concomitantly with the recognition of profound challenges in the Anthropocene including climate change, biodiversity loss, land use change, alteration of nitrogen and phosphorus cycles, and the depletion of freshwater [106]. Our ability to address these challenges depends greatly on how well we can make decisions despite great uncertainty. Although utilizing a variety of approaches is certainly of value [107], ABMs will likely play an important role in these efforts. Our intent, in utilizing a quantitative UA-SA approach, was to expand ABMs explanatory and exploratory potentials, contributing both to scientific efforts to increase our knowledge and predictive abilities and to policy requirements of making good decisions without complete knowledge.

## Supporting Information

Code S1 **Pseudo code of the main routine in the Agent-Based Model of Participation in the Conservation Reserve Program.** CRP: Conservation Reserve Program, FALLOW: total area converted to fallow (in pixels), FSA: Farm Service Agency, OWA: ordered weighted averaging decision rule, SITE: a pixel belonging to a given farm parcel. Factors are presented using uppercase bold fonts.
(DOCX)
