# Supplementary material for: Using Uncertainty and Sensitivity Analyses in Socioecological Agent-Based Models to Improve Their Analytical Performance and Policy Relevance
Source: PLoS One. 2014 Oct 23;9(10):e109779. doi: 10.1371/journal.pone.0109779 (PMC4207681; doi:10.1371/journal.pone.0109779)
Supplement: Code S1 — Pseudo code of the main routine in the Agent-Based Model of Participation in the Conservation Reserve Program. CRP: Conservation Reserve Program, FALLOW: total area converted to fallow (in pixels), FSA: Farm Service Agency, OWA: ordered weighted averaging decision rule, SITE: a pixel belonging to a given farm parcel. Factors are presented using uppercase bold fonts. (DOCX) [file pone.0109779.s001.docx]

**Code S1**

Initialize FALLOW to zero

For every year

For every farm not enrolled in CRP

Its farmer retrieves the fraction of **NEIGHBORING FARMS** enrolled in CRP (C1)

The farmer retrieves the farm's **TENURE** (C2), **VALUE OF PRODUCTION** (C3), and **FARMER's RETIREMENT** (C4)

Using a farm-specific **DECISION RULE** of OR and AND, the farmer combines C1, C2, C3, and C4 into an OWA score

If OWA score exceeds the empirical threshold

The farmer arranges, in a descending order, SITEs based on soil rental rates and environmental benefits

The farmer selects the first SITES based on his/her fraction of **LAND** to put to fallow (LOCs)

The farmer builds an offer consisting of LOCs and the rental cost

The farmer decreases the offer rental cost by **BID**

The farmer passes the offer to FSA

FSA arranges, in a descending order, the submitted offers based on their cost and the **ENVIRONMENTAL BENEFITS INDEX**

FSA selects top **N** offers to accept

For every accepted offer

For every LOC

Its farmer converts LOC's land use to fallow

The farmer adds one to FALLOW

The farmer flags farm as enrolled in CRP

Print the final FALLOW
